# LesionNet: an automated approach for skin lesion classification using SIFT features with customized convolutional neural network

**DOI:** 10.3389/fmed.2024.1487270

**Published:** 2024-10-21

**Authors:** Sarah A. Alzakari, Stephen Ojo, James Wanliss, Muhammad Umer, Shtwai Alsubai, Areej Alasiry, Mehrez Marzougui, Nisreen Innab

**Affiliations:** ^1^Department of Computer Sciences, College of Computer and Information Sciences, Princess Nourah Bint Abdulrahman University, Riyadh, Saudi Arabia; ^2^College of Engineering, Anderson University, Anderson, SC, United States; ^3^Department of Computer Science and Information Technology, The Islamia University of Bahawalpur, Bahawalpur, Pakistan; ^4^Department of Computer Science, College of Computer Engineering and Sciences, Prince Sattam Bin Abdulaziz University, Al-Kharj, Saudi Arabia; ^5^Department of Informatics and Computer Systems, College of Computer Science, King Khalid University, Abha, Saudi Arabia; ^6^Department of Computer Engineering, College of Computer Science, King Khalid University, Abha, Saudi Arabia; ^7^Department of Computer Science and Information Systems, College of Applied Sciences, AlMaarefa University, Riyadh, Saudi Arabia

**Keywords:** skin lesion classification, computer vision, customized CNN, SIFT features, deep learning

## Abstract

Accurate detection of skin lesions through computer-aided diagnosis has emerged as a critical advancement in dermatology, addressing the inefficiencies and errors inherent in manual visual analysis. Despite the promise of automated diagnostic approaches, challenges such as image size variability, hair artifacts, color inconsistencies, ruler markers, low contrast, lesion dimension differences, and gel bubbles must be overcome. Researchers have made significant strides in binary classification problems, particularly in distinguishing melanocytic lesions from normal skin conditions. Leveraging the “MNIST HAM10000” dataset from the International Skin Image Collaboration, this study integrates Scale-Invariant Feature Transform (SIFT) features with a custom convolutional neural network model called LesionNet. The experimental results reveal the model's robustness, achieving an impressive accuracy of 99.28%. This high accuracy underscores the effectiveness of combining feature extraction techniques with advanced neural network models in enhancing the precision of skin lesion detection.

## 1 Introduction

Skin diseases, particularly skin cancer, are considered among the most critical and dangerous human cancers (1). Skin cancer comes in various forms, including melanoma, squamous cell carcinoma (SCC), basal cell carcinoma (BCC), and intraepithelial carcinoma (2). Among them, melanoma is the most aggressive very fast and invasively attacks a host and tends to metastasize early (3). Based on the 2019 year-end report of the American Cancer Society, about 7,230 die from melanoma annually, and around 96,480 are diagnosed with the disease (4). For all types of skin malignancies, the mortality rate for melanoma is approximately 1.62% (5).

The reason why melanoma skin cancer should be detected early is that there are approximately 92% chances of survival in the event it is identified at an early stage (6). One challenge researchers face is the fact that melanoma skin cancer is not easily differentiated through visual observation from other types of skin cancer. The difficult thing is that melanocytic nevi and melanoma are very similar in looks and that is why it is very hard to differentiate between them using dermoscopic images. Dermoscopy, an imaging technique that uses immersion fluid and a magnifying device, is typically used to visualize the skin's surface (7).

The layer of skin that covers humans acts as a barrier between their body and the environment. It serves as the biggest organ in the body and is responsible for control, protection, and sensation (8). Millions of individuals worldwide suffer from skin disorders, which greatly impact their quality of life and physical health (9). To find skin diseases early on, routine skin examinations are required. Recognizing skin diseases early is crucial to prevent the spread of skin cancer (10). The skin regulates body temperature, protects internal organs from germs, and serves as a sensory organ (11). Thera are three layers in human skin named as epidermis, hypodermis, and dermis. The outermost layer epidermis acts as a waterproof barrier and contains melanocytes that produce the pigment melanin that determines skin-tone. The second layer is dermis that houses hair follicles, sweat glands, and connective fibers. Connective tissue and fat make up the hypodermis. Skin diseases are any illnesses or conditions that affect these layers of the skin (12). The ABCDE rule, which was proposed by Chang et al. (13) is also utilized for the identification of skin diseases. ABCDE stands for Asymmetry, Border, Color, Diameter, and Evolution (14).

According to the American-Cancer-Society (ACS), around 1.9 million new cases of cancer diagnosed in 2021 (15). It is estimated that 1,670 Americans would lose their lives to skin cancer every day in 2021. According to Van Onselen (16), skin cancer has the most advanced treatment and outlook of any disease and is the most common type in developing nations. With 500,000 new cases reported in the United States, it is now the 19th most frequent cancer worldwide. The WHO states the every 1 among three patients diagnosed with the skin cancer disease and this disease is becoming more common worldwide (17).

Previous research has demonstrated the accuracy of teledermatology-guided referrals utilizing dermatoscopy (18), as well as how it may reduce the burden on healthcare systems and shorten wait times for critical skin cancer surgery (19). One technique that can rapidly screen a large number of patients and identify those who are most at risk is the use of automated categorization systems. This can reduce the number of unnecessary clinic visits and enable the early identification of skin cancer. Consequently, the importance of techniques used for the early diagnosis of the disease has increased. Under normal circumstances, it can be challenging to differentiate between lesioned and non-lesioned areas in photographs of melanoma skin cancer. Differentiating across these domains is a difficult task that calls for expertise (20).

Computer vision heavily relies on image processing, which includes picture preprocessing, image classification, and image segmentation. These methods are being used successfully to diagnose illnesses. Machine learning (ML) models, including decision tree (DT) and random forest (RF), were utilized by researchers to classify skin lesions (21), while Akram et al. (22) employed M-SVM to segment and recognize skin lesions. Skin lesion segmentation and classification have been done using the Adaptive Neuro-Fuzzy classifier (23).

Existing studies mainly utilize machine learning models and rely on small datasets with imbalanced class distribution. Imbalanced distribution of data for various classes can negatively impact models' performance as the models may overfit the majority class. Consequently, the models show poor performance in the minority class. This study makes use of SIFT and HoG features to find the significant features. The objective of this study is to enhance the accuracy of deep learning (DL) based diagnosis of skin lesions, surpassing the performance of existing algorithms currently in use. The study seeks to enable a swift and highly accurate diagnosis of skin lesion type. Leveraging the potential of DL for rapid image-based diagnosis, the novelty of the research lies in the proposed integration of SIFT features. The following are the major contributions of this research work:

1. This research work proposes a novel ensemble model “LesionNet” incorporating the SIFT features with a customized convolutional neural network model.
2. The proposed LesionNet model is compared with HoG features, Deep CNN features embedding, and six other supervised learning models (Random forest, MobileNet, GoogleNet, DenseNet, Recurrent neural network, ResNet, and Extreme gradient boosting) to show LesionNet's superiority among them.
3. The results of the proposed “LesionNet” are further validated using k-fold cross-validation and comparison with previously published research works.

Furthermore, the paper is structured as follows: Section 2 reviews the related work. Section 3 describes the experimental methodology. Section 4 presents and discusses the results. Finally, Section 5 concludes the research and suggests potential directions for future work.

## 2 Related work

In the past, researchers have studied skin illnesses using dermoscopic pictures and a variety of DL techniques. Although the possibility for the dermoscopic imaging system to enlarge lesions, visual examination is quite difficult because of the complex architecture of lesions. Techniques for segmenting and classifying skin diseases automatically can help solve this issue. Along with the use of a literature review, it is determined that several segmentation and classification algorithms can be used to detect skin diseases, sometimes with and sometimes without pre-processing. Devices for dermatoscopy make it possible to see skin lesions. While some writers are primarily focusing on segmentation-based approaches, others are trying to categorize skin lesions using deep learning techniques. Only a few researchers are developing methods based on both classification and segmentation. Anand et al. (11) proposed a fusion-based model for skin diseases classification using dermoscopic-images with utilization of segmentation technique.

Lambert et al. (24) provides a comprehensive review of methods for uncertainty approaches in DL models used in medical-image analysis. It covers various techniques, including Bayesian neural networks, ensemble methods, and Monte Carlo dropout, emphasizing their roles in enhancing model reliability and trustworthiness. The methods thus become important in the identification of model errors and improve interpretability for clinical decision-making, opine the authors. They articulate that the need for robust validation practices is necessary so that AI solutions can effectively and safely be deployed in health settings. In the end, the review outlines future research directions in this area of importance. This work is seminal in making medical imaging AI systems more trustworthy. In a different article (25), SkiNet proposes a DL framework for skin-lesion classification with embedded uncertainty estimation and XAI. The framework provides confidence scores with visual explanations of its predictions via techniques such as Monte Carlo dropout and integrated gradients to assist dermatologists in the diagnostic process. The study demonstrates SkiNet's effectiveness in enhancing diagnostic accuracy and clinician trust. The integration of uncertainty estimation addresses significant concerns about the reliability of AI in healthcare. This paper proposes novelty by offering a more interpretable and trustworthy AI tool for medical diagnosis, showing potential for broader applications in clinical settings.

In Khan et al. (26), a binary classification test was conducted to examine how label noise impacted CNN performance in distinguishing between nevus and melanoma. The accuracy achieved was 75.03% for dermatology and 73.80% for biopsy. In Khan et al. (27), multi-class and binary classification to the HAM10000 dataset and KCGMH dataset. They trained and validated the model using EfficientNet and DenseNet. In the KCGMH dataset, the success rate was 89.5 percent for binary classifications, 85.6 percent for seven-class classifications, and 72.1 percent for five-class classifications. In Al-Masni et al. (28), suggested a model that combines a feature selection technique with a DL model to identify skin cancer. The localization step employs the contrast stretching technique. They collected features using the DenseNet201. Training and testing were performed with an accuracy of 93.4% and 94.5%, respectively, using the ISIC2017 and ISBI2016 datasets. In Polat et al. (29), a multi-class skin lesion segmentation and classification approach was presented using a ten-layer CNN and the Deep Saliency Segmentation method. The study utilized several datasets including PH2, ISBI 2017 (2,750 images), ISIC 2018 (3,694 images), and ISBI 2016 (1,279 dermoscopic images). They also employed 10,015 dermoscopy images from the HAM10000 dataset for categorization. The accuracy achieved on these datasets were as follows: ISBI 2016—95.38%, ISBI 2017—95.79%, ISIC 2018— 92.69%, PH2—96.70%. Overall, they achieved a categorization accuracy of 90.67%. In Salian et al. (30), offered a mix of categorization and segmentation. First, a DL Full Resolution Convolutional Network (FrCN) is used for segmentation. Using transfer learning models, segmented images are classified in the following step. ISIC 2016, 2017, and 2018 were used to assess the model. In Salian et al. (31), two approaches were presented for classifying skin diseases: the first approach utilized a CNN, while the second approach combined a convolutional method with a one-versus-all strategy. The HAM10000 dataset is utilized and gives 77.7% accuracy in classifying seven skin cancer types with the CNN approach, compared to 92.9% accuracy on binary classification.

In Zafar et al. (32), melanoma was identified from images of skin lesions using both augmented and non-augmented data from the PH2 and HAM10000 datasets. In this work, the implementation of MobileNet and VGG16 models was conducted, together with a custom model developed to compare its results against the previously trained models. The accuracy rates after these works were reportedly 80.07% for VGG16 and 81.52% for MobileNet, while for a custom model, it reached 83.15%. Hauser et al. (33) designed a melanoma recognition algorithm based on CNNs that merged segmentation and classification into one process. First, they explored residual learning and proposed a two-stage framework with a convolutional residual network, which was tested using the ISBI 2016 dataset. As noted, most traditional segmentation methods lack contextual understanding; thus, this method failed to segment the objects from complicated backgrounds efficiently. To circumvent all these issues, they resorted to the U-Net architecture since it can retain the integrity of images due to the encoder-decoder pathways, which immensely aid in capturing both contextual and localization features.

It has been discussed (34), how explainable Artificial Intelligence was utilized to detect skin cancer. XAI alludes to AI models helps dermatologist for better understanding of model predictions and diagnosing. The authors identified 37 studies across various databases that applied XAI techniques for skin-cancer diagnosis. They discussed methods like rule-based models, gradient-based strategies, and decision trees, highlighting XAI's limitations, alongside benefits like improved transparency. The study concludes that XAI could enhance skin lesion detection by offering understandable and transparent models, but further research is needed to integrate these models into healthcare systems effectively and validate their clinical utility.

In Bhatt et al. (35), provided a critical evaluation of a few state-of-the-art ML techniques for skin lesion diagnosis. The scientists additionally highlighted the importance of early identification of melanoma skin cancer because it leads to much higher survival rates. The authors thoroughly covered a wide range of topics, including feature extraction techniques, data augmentation, and various models like KNN, CNN, and SVM. All of which were applied to datasets compiled from the ISIC and ISBI archives. Nonetheless, the work was subject to certain limitations. Bias in the training set and other potential disadvantages of applying ML algorithms to the diagnosis of melanoma were not discussed in the study. The authors also did not explore the possible moral implications of utilizing machine learning algorithms for medical diagnosis.

## 3 Materials and methods

This section gives details about the dataset, dataset preprocessing (data-augmentation), models employed for the detection of skin-lesion, and the proposed methodology of “LesionNet” also discussed in this section. This section also discusses all seven evaluation metrics utilized in this research work.

Multiple methods can be used to diagnose skin diseases. From these multiple methods, we have utilized dermoscopic image-based detection because it is the most effective one. The rest of the methods are as follows:

- Dermatoscopy is the initial process it involves using a dermatoscopy instrument to check out the blood vessel and pigmentation of a mole without removing it. Patch tests, skin biopsies, and cultures are the most popular types of skin tests (11).
- While automated dermatoscopic image analysis, particularly using neural networks, has been researched for many years (36), it is now growing in popularity and has shown encouraging results when compared to medical professionals (37).
- A neural network can also be trained to analyze clinical close-up (macroscopic) photos to diagnose skin cancer; however, this method has been shown to have lesser accuracy when predicting various disease classifications (38).

### 3.1 Dataset

**Figure 1:**
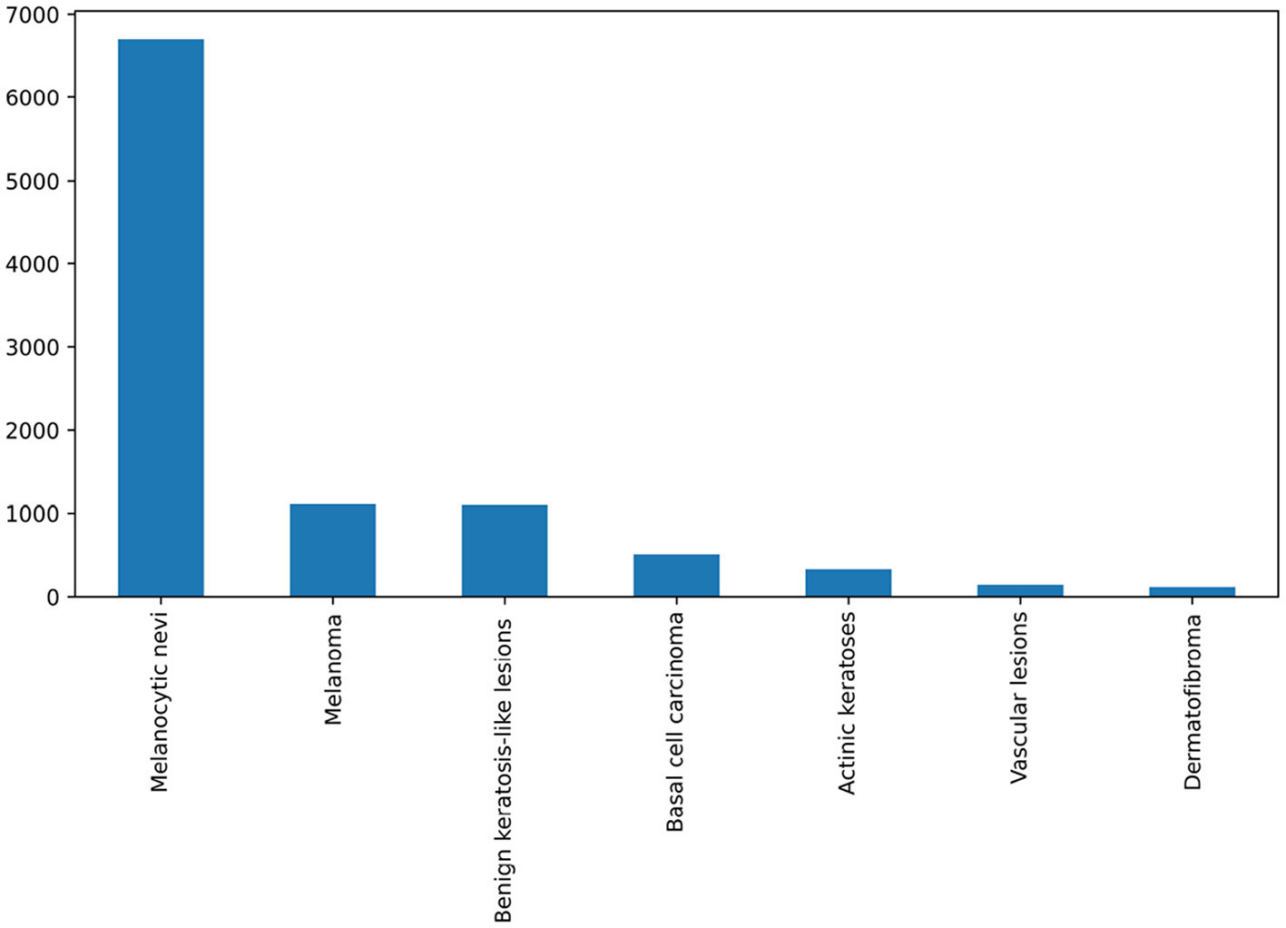
Count of each skin lesion type in dataset.

**Figure 2:**
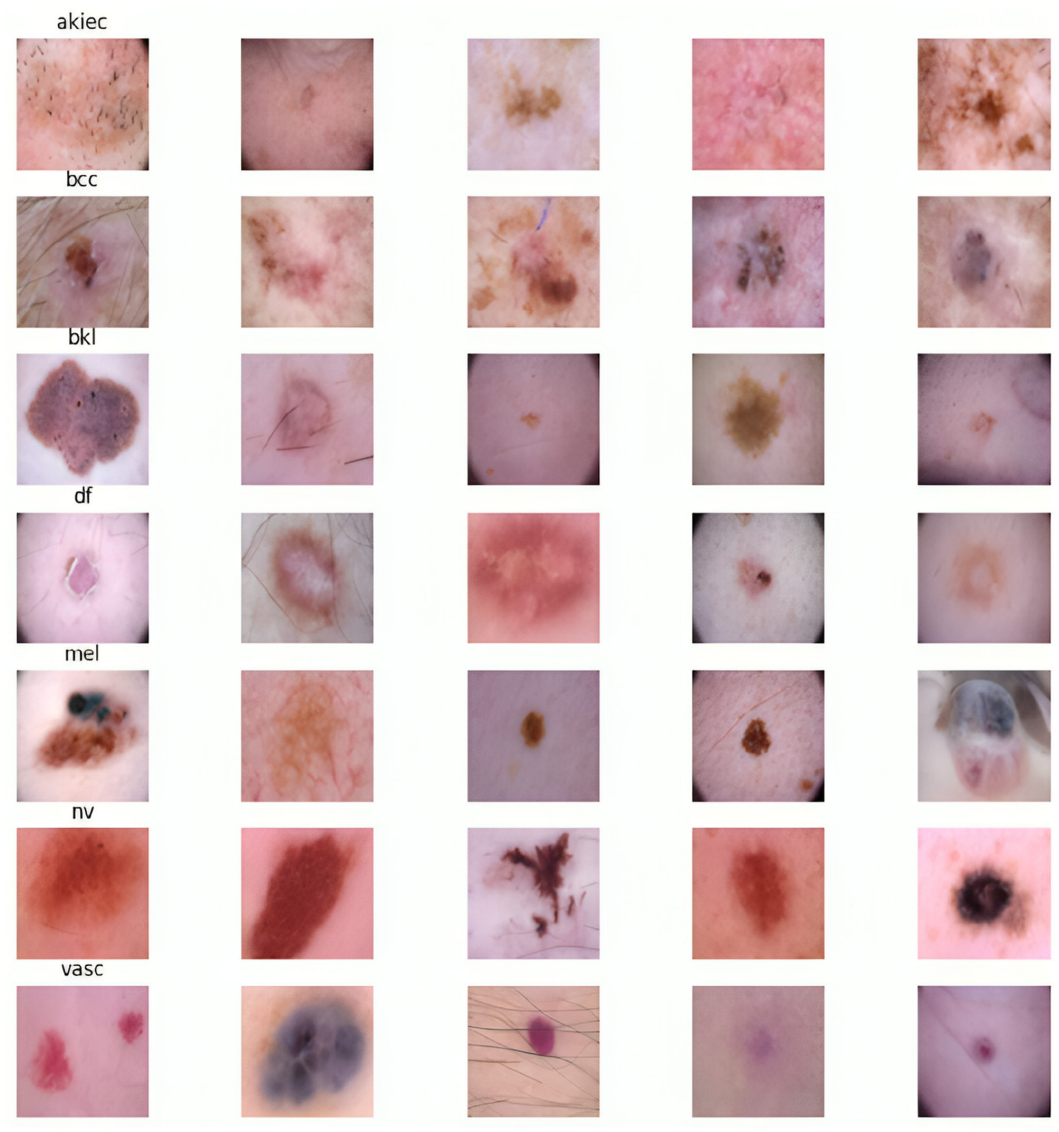
Example images of each skin lesion type in dataset.

In this research work, we have utilized a benchmark dataset for ensuring reliability and trustworthiness (23). It consists of 10,000 skin images from different patients, meticulously categorized into seven distinct types, which serve as ground truth labels for classification purposes. Figure 1 illustrates the distribution of each class within the dataset, while Figure 2 displays sample images from each skin cancer type. Notably, the dataset includes a significant portion of nearly 68,000 images. Recognizing the clinical relevance of cancer location in diagnosis and treatment, the dataset encompasses images depicting various body parts.

### 3.2 Image data augmentation

An efficient and effective CNN model is achieved when the validation error decreases gradually along with the training error, leading to improved accuracy rates. Increasing the amount of image data is a potent technique to address this challenge, as it enlarges the dataset. This augmentation enhances the model's ability to generalize and improves its accuracy. Training CNNs on extensive datasets is crucial for boosting accuracy by enhancing generalization capabilities. Therefore, tools like the image generator provided by Keras are utilized for data augmentation. This generator offers options such as rotation range, width shift range, zoom range, and height shift range to augment data. Table 1 illustrates the effect of image augmentation.

**Table 1:** Effect of image data augmentation on each skin lesion type.

**Classes**	**Lesion before augmentation**	**Lesion after augmentation**
Melanoma	1,000	3,000
Melanocytic Nevi	6,800	7,000
Basal-cell-carcinoma	500	2,000
Benign-keratosis-like-lesions	1,000	3,000
vascular-lesions	225	2,000
Actinic-keratoses	250	2,000
Dermatofibroma	225	2,000
Total lesion images	10,000	21,000

### 3.3 Image feature extraction techniques

#### 3.3.1 Scale-invariant feature transform (SIFT)

Since its introduction, SIFT has been one of the most widely used and successful approaches for image feature extraction, given its invariance to changes in scale, orientation, or illumination (39). First proposed in 1999 by David Lowe, SIFT detects key points in images, generally around conspicuous regions, and describes their local neighborhood using histograms of gradient directions. These descriptors are invariant concerning changes in scale and orientation and, hence, very suitable for matching key points across different images. Apart from that, SIFT has been applied to object recognition, image stitching, and 3-D reconstruction. While SIFT is very effective, the high dimensional feature vectors can lead to low computational efficiency. Its strength and flexibility support its continuance as an essential tool in computer vision research and applications. Let's understand how SIFT feature extraction works with an example: Let *I*(*x, y, c*) be the RGB skin lesion image, with (*x, y*) pixel coordinates and color channel *c*representing Red, Green, or Blue.

##### 3.3.1.1 Cumulative feature extraction process

1. **Convert the RGB image to grayscale:**


(1) $$\begin{array}{l} I_{\text{gray}}\left(x,y\right)=0.2989\cdot I\left(x,y,\text{R}\right)+0.5870\cdot I\left(x,y,\text{G}\right)+0.1140\cdot I\left(x,y,\text{B}\right) \end{array}$$

where *I*_gray_(*x, y*) is the grayscale image.

2. **SIFT feature extraction:**

- **Scale-space extrema detection:**

(2) $$\begin{array}{l} K_{i}=\arg\underset{\left(x,y,\sigma \right)}{\max}|L\left(x,y,k\sigma \right)-L\left(x,y,\sigma \right)| \end{array}$$

where *L*(*x, y*, σ) is the scale-space representation obtained by convolving the grayscale image *I*_gray_ with a Gaussian kernel of scale σ.

- **Keypoint localization:** Refine the key points by fitting a quadratic function to the local sample points.
- **Orientation assignment:**

(3) $$\begin{array}{l} \theta_{i}=\arg\underset{\theta }{\max}\underset{\left(x,y\right)\in \text{neighbor}\left(K_{i}\right)}{\sum }|\nabla I_{\text{gray}}\left(x,y\right)|\cdot \delta \left(\theta -\theta \left(x,y\right)\right) \end{array}$$

- **Descriptor computation:**

(4) $$\begin{array}{l} D_{i}=\left[H\left(\theta_{1}\right),H\left(\theta_{2}\right),\ldots ,H\left(\theta_{n}\right)\right] \end{array}$$

where *H*(θ_*j*_) is the histogram of gradient orientations within the keypoint's neighborhood.

#### 3.3.2 Histogram of oriented gradients (HoG)

Among the most pervasive feature descriptors in computer vision applications like object detection and image classification is the HoG descriptor (40). HoG was first presented in 2005 by Navneet Dalal and Bill Triggs, and the method works by partitioning an image into small spatial regions called cells and then computing the histograms of gradient orientations within each cell. These histograms thus summarize the distribution of edge orientations and, therefore, encode compactly the local image characteristics. HoG generates a descriptor resilient to changes in illumination and resistant to local geometric distortions by aggregating histograms over several cells and optionally normalizing them. Such success has been realized in applications of pedestrian detection, face detection, and gesture recognition using the HoG. While this is very effective, HoG may struggle with complex background clutter and occlusion scenarios. Nevertheless, simplicity and robustness make it a great tool in many computer vision systems. Let's understand how HoG feature extraction works with an example:

#### 3.3.3 HoG feature extraction

- **Gradient computation:**

(5) $$\begin{array}{l} \nabla I_{\text{gray}}\left(x,y\right)=\left(\frac{\partial I_{\text{gray}}}{\partial x},\frac{\partial I_{\text{gray}}}{\partial y}\right) \end{array}$$

- **Orientation binning:**

(6) $$\begin{array}{l} H_{c}\left(\theta \right)=\underset{\left(x,y\right)\in c}{\sum }|\nabla I_{\text{gray}}\left(x,y\right)|\cdot \delta \left(\theta -\theta \left(x,y\right)\right) \end{array}$$

- **Normalization:**

(7) $${\hat{H}}_{b}=\frac{H_{b}}{\sqrt{\|H_{b}{\|}^{2}+\epsilon^{2}}}$$

### 3.4 Deep learning models

To classify skin lesions, this research suggested a deep technique based on many pre-trained convolutional neural networks and picture super-resolutions.

#### 3.4.1 DenseNet201

Probably the most typical property of the DenseNet model architecture is its dense connectivity pattern. Each layer has direct connections to every subsequent layer (41). In the design, multiple-layer feature reuse enhances computational efficiency and allows more diversified input into the network for later layers. This design encourages feature aggregation during the process of model learning. In DenseNet, every layer receives all previous layers' feature maps, which allows for the reuse of the features and has been previously proven to ease gradient flow. For the improvement of the efficiency of the method and model complexity reduction, DenseNet implements bottleneck layers and pooling layers in the transition layer such (42). It's somehow close to what is applied in the principles of ResNet designs. However, unlike ResNet, where each layer receives input directly from the previous layer, DenseNet diverges by connecting each layer densely in a feed-forward manner, ensuring comprehensive feature utilization and integration.

#### 3.4.2 GoogleNet

The network designed in 2015, known for its comprehensive approach in CNN models, is based on GoogleNet architecture (43). GoogleNet employs inception modules consisting of 1 × 1, 3 × 3, and 5 × 5 convolutional sub-layers to extract features of various sizes and concatenate them for the subsequent layers. These modules operate in parallel and incorporate 3 × 3 maximum pooling layers (44) to process data received from preceding layers. To optimize computational efficiency, a 1 × 1 convolution is applied with max-pooling layer. Each segment of the inception module computes unique features and their outputs are merged and fed as inputs to subsequent CNN layers. Instead of fully connected layers, starter modules are used in this architecture. GoogleNet employs maximum pooling at certain stages to reduce information volume from critical layers, and it concludes with an average pooling layer at the network's end.

#### 3.4.3 MobileNetv2

To address the problem of information extinction in nonlinear layers within convolutional blocks, this network employs linear bottlenecks and significantly separable convolutions (DSC), as described in Yilmaz et al. (45). To maintain information, it also presents an entirely new structure known as inverse residuals. Deep, separable convolution is the foundation of the MobileNet architecture. Each input channel undergoes standard convolutional processing, followed by depth-wise inversion, and concludes with a final convolution of all input channels with the filter channel. As a result, a filtered output channel is produced. Next, these channels are piled up. These channels are combined into a single channel using 1 × 1 convolution in deep convolution. Therefore, it is known that this method decreases the number of parameters and boosts efficiency even if it produces the same outputs as normal convolution (46).

#### 3.4.4 Recurrent neural network (RNN)

A neural network designed for sequential processing, where components from one iteration feed into the next, is known as a RNN. In RNNs', hidden layers from previous iterations contribute input to the same hidden-layer and to subsequent iterations. This capability allows RNNs to learn from past attempts on earlier segments of sequences, making them highly effective for sequence evaluation (47).

#### 3.4.5 Convolutional neural network (CNN)

CNN is like a smart system with different layers. The first layer helps pick out important details, the next layer makes things smaller, another layer prevents it from getting too focused on certain details, and the last one turns everything into a neat arrangement. They use something called ReLU to make it all work, and there's a dropout layer that helps with not getting too caught up in the details, set at a rate of 0.2 in this study (48).

### 3.5 Machine learning models

#### 3.5.1 Random forest (RF)

RF is a meta-estimator that enhances success and mitigates overfitting by aggregating data from multiple DT (49). Each DT classifier in RF is trained on a subset of input data samples. The ensemble then averages the outputs of these decision trees, functioning as a collective learner. RF generates a large number of DT, where each tree predicts the mean for regression tasks or the output class for classification at each node. It is a versatile and widely used machine learning algorithm known for its ability to produce reliable results without extensive hyperparameter tuning.

#### 3.5.2 Extreme gradient boosting (XGBoost)

XGBoost is a robust ML algorithm that has gained widespread popularity for its efficiency and effectiveness in various predictive modeling tasks (50). Introduced by Chen and Guestrin in 2016, XGBoost is highly scalable, efficient, and adaptable. XGBoost enhances traditional gradient boosting with features like a regularized learning objective to prevent overfitting, support for parallel and distributed computing, and an innovative tree construction algorithm that maximizes performance. These attributes make XGBoost well-suited for handling large-scale datasets and achieving state-of-the-art results in tasks such as classification, regression, and ranking.

### 3.6 Proposed LesionNet framework

**Figure 3:**
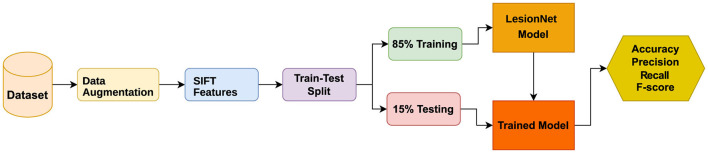
Proposed methodology diagram.

CNN is a special tool used mainly for sorting out pictures into different categories (51). It's made up of layers that are good at dealing with 2D or 3D data, especially when it comes to visual images. The proposed LesionNet framework (as shown in Figure 3) is based on the usage of SIFT features with a customized (layer-wise arrangement) CNN model. Think of it like having different layers where the first one looks at the picture, and the others learn more and more about it. They use something called ReLU to help with the learning process The complete layer-wise structure of the proposed LesionNet model is shown in Table 2. CNN needs at least one convolutional layer because it works by looking at parts of the picture instead of the whole thing. It has input and output layers, and in the middle, there are hidden layers that do a lot of the work. These hidden layers have different jobs, like making the picture simpler or figuring out what's important. In this study, we also use a max-pooling layer to help CNN do an even better job.

**Table 2:** Layer-wise structure of proposed LesionNet model.

**Name**	**Description**
Convolutions	Filters=(@4, @32), Strides=(@2)
Convolutions	Filters=(@4, @32), Strides=(@2)
Max-Pooling	Pool-size=(@2), Strides=(@2)
Convolutions	Filters=(@4, @32), Strides=(@2)
Average-Pooling	Pool-size=(@2), Strides=(@2)
Layers	Flattened()
Fully-Connected	Dense(180-neuron)
Fully-Connected	Dense(120-neuron)
Fully-Connected	Dense (32neuron)
Sigmoid	Sigmoid(2)

#### 3.6.1 Max pooling layer

In deep neural networks, they use a process called max pooling to simplify information (52). It's like picking out the most important parts. Some of the most important goals of max-pooling are those designed to make output features from the hidden layers smaller and, therefore, more efficient. Downsizing in this way dispenses with large amounts of data, hence becoming more straightforward to handle, avoiding getting too caught up in details, which may help prevent overfitting. By doing so, they reduce the number of things the system is thinking about to a high degree, therefore making it run faster. Think of it like looking at some big picture and focusing on its essential parts to understand it better.

#### 3.6.2 Fully connected layer

Fully connected layers are vital parts of CNN, especially in the analysis and classification of skin images. In CNNs, the model breaks down image data into smaller sub-components referred to as feature maps, all going through independent learning processes. The feature maps capture essential details from the image and are then passed on to fully connected layers. Neurons are small units interconnecting with other neurons in these layers through weighted connections that process information. They all work together to classify the images of skin cancer, including NV, bkl, MEL, BCC, vasc, akiec, and df. This is done by dropping some connections that seem redundant in the neurons, thereby increasing the accuracy and speed of decision-making. This teamwork of neurons summarizes understanding the input image and then classifying it correctly.

#### 3.6.3 Dropout rate

The dropout layer may be thought of as a valuable tool in neural networks. This approach is used to prevent the system from being too good at training data and overthinking. It does this by randomly turning off some of the little units, or neurons, over the network using a kind of random choice. By doing so, it enables the neural network not to get too mired with details and allows it to focus on essential things. Imagine that all of the little thinking units were on all of the time; then, the network would learn too much from some specific details, and that would be a problem when presented with new data. The dropout layer says, “Hey, let's turn off some units now and then so we get a more balanced and useful understanding of the data.” It is one of those nice little tricks that make neural networks learn well and not focus on things that may not be important.

#### 3.6.4 Sigmoid

The simplest would be a linear activation function, but its disadvantage is that it will be able to handle only simple patterns. Any neural network made of linear functions is easily trainable but may not learn complex features efficiently. These make it easier for neurons to learn complex patterns. The sigmoid function also called the logistic-function, gives an output between 0.0 and 1.0, which aids information presentation for better understanding. In medical image analysis, using CNN-based DL techniques can be very useful because they automatically identify the prominent features from the images to classify them into respective categories. However, their training process is tricky, especially when there is a lack of large data sets. The problems of overfitting and underfitting can occur. This paper addresses this challenge through several techniques to ensure robust learning from limited data.

### 3.7 Evaluation parameters

The metrics generally used to evaluate DL models are recall, F1 score, precision, and accuracy. Based on multi-class classification problem, additional metrics include AUC, MCC, and Kappa. Formulas for all evaluation metrics are:


(8) $$\begin{array}{l} Recall=\frac{TP}{TP+FN} \end{array}$$

(9) $$\begin{array}{l} Precision=\frac{TP}{TP+FP} \end{array}$$

(10) $$\begin{array}{l} Accuracy=\frac{TP+TN}{TP+TN+FP+FN} \end{array}$$

(11) $$\begin{array}{l} F1score=2\times \frac{Precision\times Recall}{Precision+Recall} \end{array}$$

The AUC measured the quality of model predictions from zero to one, where one is the best and 0 is the worst. Moreover, AUC reflects the degree of separability that explains how healthy classes are distinguishable by the model. The Kappa is a statistics-based prominent evaluation metric denoted by k, measuring the reliability of other evaluators or parameters. The following equation computes the value of k:


(12) $$\begin{array}{l} k=\frac{prob\left(O\right)-prob\left(C\right)}{1-prob\left(C\right)} \end{array}$$

Matthews correlation coefficient (MCC) is an unaffected and substitute measure for uneven datasets and employs a likelihood matrix method to calculate the Pearson product-moment correlation coefficient between predicted and actual values (53). Expressed in terms of the entries of the contingency matrix M, MCC is formulated as follows:


(13) $$\begin{array}{l} MCC=\frac{TP.TN-FP.FN}{\left(TP+FP\right).\left(TP+FN\right).\left(TN+FP\right).\left(TN+FN\right)} \end{array}$$

## 4 Results and discussions

The Python 3.8 with supporting libraries TensorFlow and Scikit-learn is utilized for experimentation. The experimental setup operated with 8GB of available RAM, on Windows 10 (64-bit) operating system. The CPU utilized was 7th Gen, Intel Core i7 having a clock speed of 2.8 GHz, and an 8 GB, GTX 1060 GPU from Nvidia. Technical specifications of the computational resources deployed in research are crucial for an in-depth understanding of the model. Table 3 summarizes the experimental setup.

**Table 3:** Framework specifications.

**Aspect**	**Details**
Programming environment	Python 3.8
Software libraries	Scikit-learn, TensorFlow
Operating system	Windows-10
Processors	7th Gen, Intel-Core i7, 2.8 GHz processor
Graphic card	8 GB- GTX 1060 Powered GPU from Nvidia

### 4.1 Results of all supervised learning models on the skin lesion dataset using SIFT features

**Figure 4:**
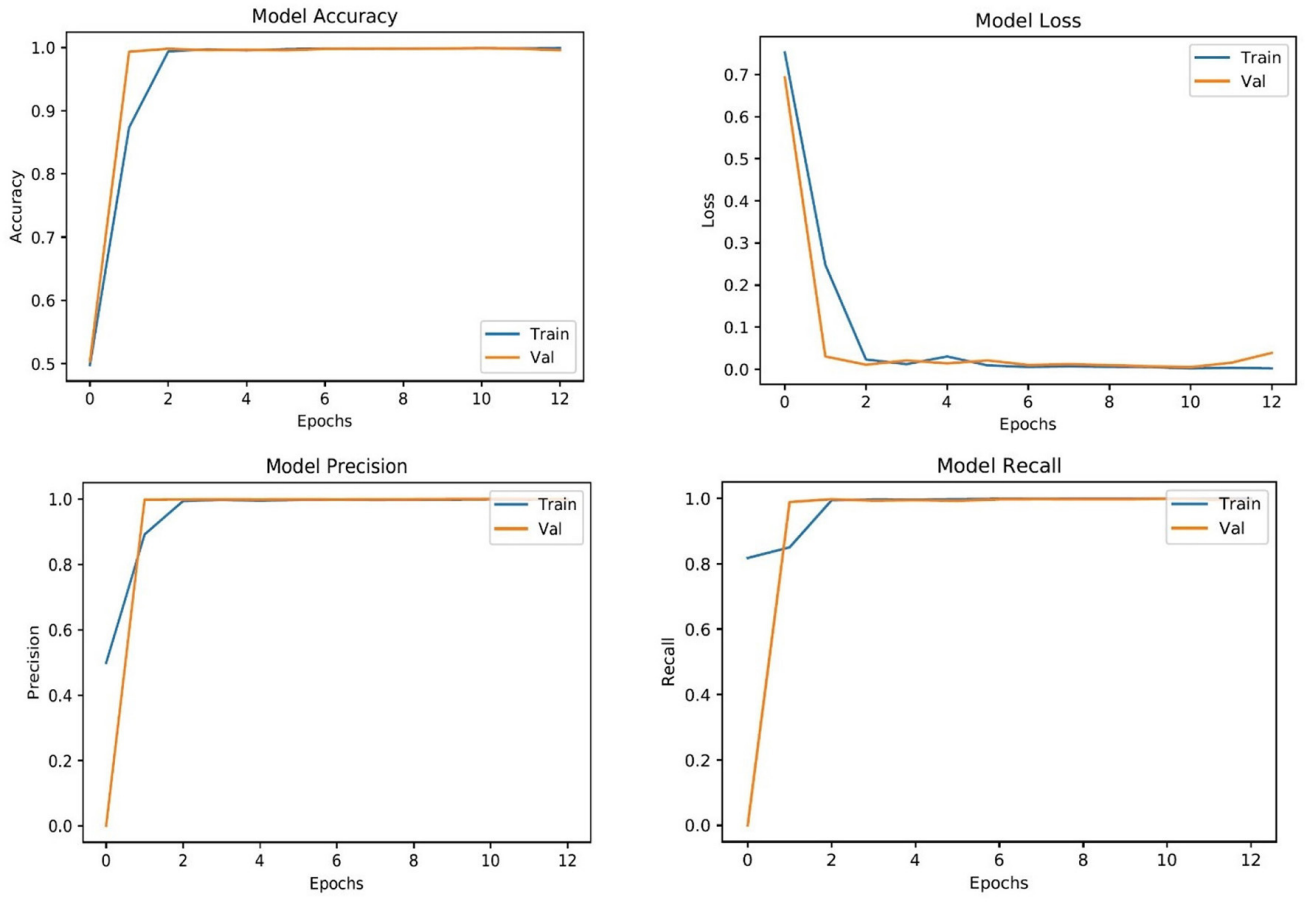
Training and testing validation curves.

The complete classification report of all supervised learning models is shown in Table 4. As can be seen in Table 4, CNN perform better than MobileNet in general because of their advanced architecture and range of capabilities. CNNs use several layers of convolution and pooling techniques to capture complex patterns of space in images. They can extract more abstract properties from the input data thanks to these procedures, which is essential for applications like object detection and image classification. However, MobileNet is designed with efficiency in mind, especially for mobile and embedded devices, thanks to its use of depthwise separable convolutions, which significantly decrease computing costs. MobileNet provides an attractive trade-off between speed and accuracy, making it appropriate for limited resource applications, even though it compromises some performance when compared to typical CNNs. However, because of their deeper structures and capacity to recognize more complex patterns, CNNs often do very well in jobs where accuracy is crucial. Therefore, the decision between CNNs and MobileNet is based on the particular needs of the application, taking into consideration factors like required accuracy, speed, and processing resources (54). To show the smooth training and testing of the proposed LesionNet framework, we have added the validation curves that show in Figure 4 that model training is smooth and does not get any underfitting or overfitting.

**Table 4:** Classification report of all supervised learning models.

**Model**	**Acc**.	**AUC**	**Rec**.	**Prec**.	**F1 score**	**Kappa**	**M.C.C**.
LesionNet	0.9928	0.9995	0.9928	0.9930	0.9928	0.9892	0.9893
Random Forest	0.9712	0.9982	0.9712	0.9720	0.9714	0.9568	0.9571
MobileNet	0.9820	0.9985	0.9820	0.9820	0.9820	0.9730	0.9730
GoogleNet	0.9784	0.9988	0.9784	0.9787	0.9785	0.9676	0.9678
DenseNet	0.9892	0.9999	0.9892	0.9892	0.9892	0.9838	0.9838
RNN	0.9784	0.9973	0.9784	0.9786	0.9784	0.9676	0.9677
ResNet	0.9856	0.9997	0.9856	0.9858	0.9855	0.9783	0.9785
XGBoost	0.9603	0.9983	0.9603	0.9608	0.9601	0.9404	0.9409

Because CNN can learn structures directly from raw input, such as photos, without depending on handcrafted features, they often perform better than Random Forests. CNNs use convolutional and pooling layers to continuously extract information at multiple levels of abstraction, which makes them excellent at tasks like object detection and image categorization. CNNs can recognize intricate patterns and correlations in the data because of these learned features that are maximized during training, which is especially useful for jobs requiring high-dimensional input, like picture analysis. Conversely, Random Forests are a method of ensemble learning based on decision trees that work by dividing the feature space into smaller regions and predicting the target variables based on their mean (regression) or mode (classification) within those regions. Although Random Forests work well for many machine learning applications, they frequently have trouble with high-dimensional data and may need a lot of feature engineering to perform as well as CNNs. CNNs are also more flexible and able to capture subtle associations because they can automatically adjust to the underlying structure of the input, which is useful in challenging picture identification applications (55).

CNNs generally surpass GoogleNet, also known as Inception, for several reasons. While GoogleNet introduced inception modules to optimize computational resources by employing multiple convolutional filter sizes within a single layer, CNNs typically outperform it due to their deeper architectures and larger parameter space. The depths of CNN enable it to do marvelously well in capturing complex hierarchical features in data, especially in image classification and object detection. By using convolution, pooling, and nonlinear activation functions across multiple layers, a CNN learns increasingly abstract representations of input data. This allows the modeled capture of intrinsically complex patterns and relationships within the data and thus often yields better performance than GoogleNet in many scenarios. Although GoogleNet was, in its time, very innovative and had proposed some cool ideas about the improvement of computational efficiency in DL models, subsequent improvements in CNN architectures have often outperformed it (56).

Due to the dissimilar nature of architectural designs and their enhanced ability concerning collect hierarchical features, CNNs usually perform better than DenseNet. Although DenseNet creates dense connections across stages to connect each layer to every other layer, aiming at reusing features and resolving the vanishing gradient problem, CNNs with conventional architectures have often excelled in many applications compared with DenseNet. CNNs, due to the application of convolutional and pooling layers, have a particular strength in object detection and image classification. These networks are designed to learn representations of the input data at different levels of abstractions, a hallmark for applications with high-dimensional inputs like picture analysis. Moreover, in most cases, deeper CNNs are better than DenseNet simply because of the deeper design that lets them recognize more and more complex patterns and correlations within the data.

Since CNNs are good at capturing spatial hierarchies and local patterns in data, they often work better compared to recurrent neural networks for certain tasks such as detection and image categorization. They are designed especially to process grid-structured data like photographs. It achieves this through local connectivity and shared weights performing pooling and convolutions, extracting features from the input itself. These techniques enable CNNs to automatically learn hierarchical representations directly from the raw data, which may turn very useful in piling jobs that require inputs of high dimensions, as in picture analysis. However, RNNs perform better with sequential data in applications like time series analysis and natural language processing where the sequence of the inputs is important. RNNs may have trouble representing the spatial links found in grid-like structures like pictures, even though they are excellent at modeling time-dependent and sequential patterns. Consequently, CNNs typically outperform RNNs in applications where spatial information is critical, including picture categorization (54).

CNNs, especially deep architectures such as ResNet, frequently achieve superior performance compared to shallower CNNs because they effectively mitigate the vanishing gradient problem and facilitate the training of extremely deep networks. ResNet introduced skip connections, also known as residual connections, which enable the network to learn residual functions relative to the inputs of each layer. This approach simplifies the optimization process for very deep networks. Despite the success of ResNet in various tasks like image classification and object detection, traditional CNNs can still outperform ResNet in certain scenarios. This can happen when the dataset is not sufficiently complex to necessitate the depth of ResNet or when computational resources are limited. Additionally, for tasks where interpretability is crucial, shallower CNNs might be preferred over ResNet due to the difficulty in interpreting the learned representations in very deep networks. Therefore, while ResNet offers significant advantages in terms of training deep networks, the choice between ResNet and traditional CNNs depends on the specific requirements of the task at hand (57).

Since CNNs can automatically build hierarchical representations from raw data, they frequently perform better than XGBoost, especially in tasks like identifying objects and image classification. Through convolutions and pooling processes, CNNs are particularly good at catching local patterns and spatial hierarchies in data, which helps them extract ever more abstract features from the input data. CNNs can recognize complicated relationships and trends in the data because these learned features are maximized during training, which is crucial for tasks requiring high-dimensional input, including picture analysis. XGBoost, on the other hand, is an ensemble learning technique based on decision trees. It works by repeatedly dividing the feature space and generating predictions using the combined judgment of several weak learners. Though XGBoost excels at many machine learning tasks, such as structured data analysis and tabular data analysis, its inability to automatically learn hierarchical representations makes it less suitable for high-dimensional data, such as photos. Consequently, CNNs typically outperform XGBoost in situations where capturing complex spatial relationships and patterns is essential (58).

### 4.2 Results of all supervised learning models on the skin lesion dataset using HoG features

**Figure 5:**
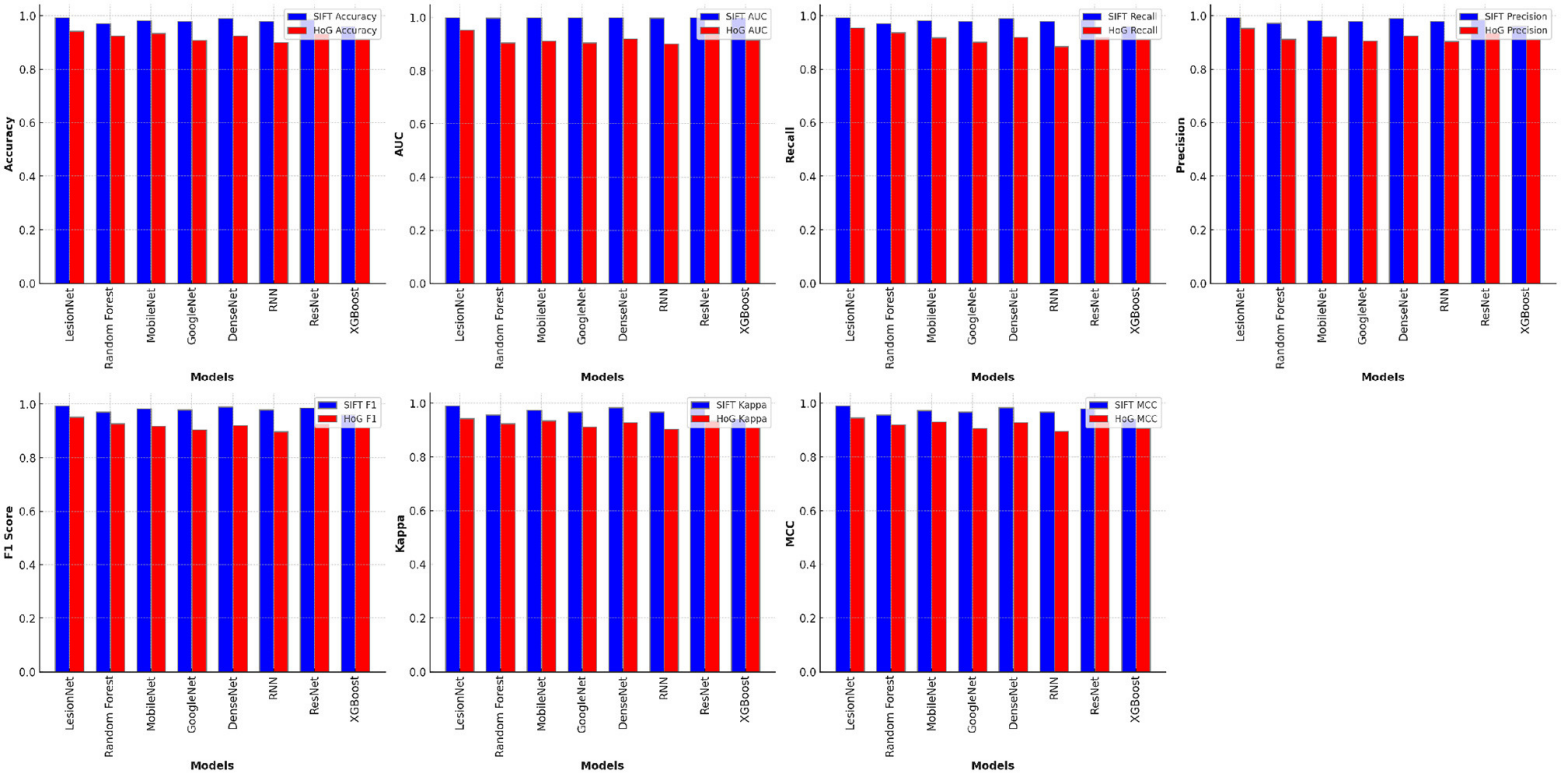
Comparison of all model results using SIFT and HoG features.

In Table 5, it can be observed that all supervised learning models' performance is affected using HoG features. The performance of all learning models decreases, especially recurrent neural networks. The complete comparison of all model results using SIFT and HoG features is shown in Figure 5.

**Table 5:** Classification report of all supervised learning models.

**Model**	**Acc**.	**AUC**	**Rec**.	**Prec**.	**F1 score**	**Kappa**	**M.C.C**.
LesionNet	0.9415	0.9528	0.9544	0.9531	0.9511	0.9434	0.9461
Random Forest	0.9248	0.9048	0.9364	0.9125	0.9264	0.9232	0.9194
MobileNet	0.9344	0.9114	0.9169	0.9224	0.9186	0.9341	0.9294
GoogleNet	0.9068	0.9057	0.9007	0.9063	0.9048	0.9112	0.9066
DenseNet	0.9242	0.9194	0.9196	0.9242	0.9212	0.9276	0.9290
RNN	0.8996	0.9014	0.8856	0.9032	0.8969	0.9031	0.8956
ResNet	0.9322	0.9356	0.9197	0.9367	0.9255	0.9344	0.9316
XGBoost	0.9125	0.9165	0.9116	0.9116	0.9116	0.9094	0.9059

### 4.3 Results of all supervised learning models on the skin lesion dataset using deep convolutional features

The third step of experiments contains the results of all learning models utilizing CNN features. The results of all learning models are shared in Table 6. From the results, it can be concluded that the results of CNN embedding features are better than HoG features but still SIFT features results are above all in terms of all evaluation metrics.

**Table 6:** Classification report of all learning models utilizing deep CNN features.

**Model**	**Acc**.	**AUC**	**Rec**.	**Prec**.	**F1 score**	**Kappa**	**M.C.C**.
LesionNet	0.9634	0.9752	0.9798	0.9647	0.9733	0.9591	0.9597
Random Forest	0.9527	0.9456	0.9517	0.9581	0.9547	0.9464	0.9487
MobileNet	0.9498	0.9453	0.9328	0.9474	0.9410	0.9443	0.9419
GoogleNet	0.9518	0.9498	0.9534	0.9596	0.9558	0.9509	0.9458
DenseNet	0.9460	0.9398	0.9447	0.9393	0.9444	0.9387	0.9319
RNN	0.9236	0.9242	0.9002	0.9014	0.9012	0.9086	0.9067
ResNet	0.9418	0.9385	0.9214	0.9325	0.9291	0.9435	0.9402
XGBoost	0.9219	0.9185	0.9227	0.9252	0.9239	0.9190	0.9209

### 4.4 Results of proposed LesionNet model using all seven classes

In this subsection, we have tested our proposed LesionNet model onthe complete dataset in which all target classes augmented training dataset is utilized for training. The dataset augmented dataset details are shared in Table 1. The proposed LesionNet model performs very well even in the case of multi-class classification and gives an overall accuracy of 90.12%. The class-wise results of all target class is shared in Table 7.

**Table 7:** Class-wise accuracy results using LesionNet model.

**Target class**	**Accuracy**	**Overall accuracy**
Melanoma	88.36%	90.12%
Melanocytic Nevi	94.67%		
Basal cell carcinoma	89.47%		
Benign keratosis like lesions	87.36%		
vascular lesions	89.96%		
Actinic keratoses	89.35%		
Dermatofibroma	91.68%		

### 4.5 Significance of proposed LesionNet model

To check the stability and significance of the proposed LesionNet model, we have trained and tested it on another independent dataset named “SIIM-ISIC Melanoma Classification” (59) International Skin Imaging Collaboration (ISIC). The proposed LesionNet model gives an accuracy of 96.67%, 95.49% precision, 97.24% recall, and 96.32% F1 score. These results confirm that the proposed model is a reliable framework for medical skin lesion diagnosing and predicting the types of skin cancers.

### 4.6 Discussion on the LesionNet performance

LesionNet performance is best when we extract features using SIFT. The motivation to use SIFT features for skin lesion classification is multifaceted. SIFT is inherently designed to be invariant to changes in scale and rotation, which is crucial for medical images where lesions may appear at different sizes and orientations. In addition, the SIFT features are invariant to illumination changes and viewpoints; thus, this augments the resilience of the classification model. The items of interest of SIFT are the identification and description of locations of key points within images, facilitating associated critical texture and shape details of lesions. The inclusion of SIFT features in CNN models enhances performance by providing robustness to scale, rotation, and noise. SIFT extracts keypoints that capture fine details and textures, improving classification accuracy, especially in complex images like skin lesions. This leads to better generalization across datasets and reduces sensitivity to image variations, where standard CNNs may struggle. By focusing on strong edges and local patterns, SIFT enhances precision, making the model more effective at distinguishing between different lesion types. Overall, combining SIFT with CNNs offers a richer and more resilient feature set than standard CNNs alone.

In addition, SIFT is highly efficient in terms of computational performance and proves to be a practical method, demanding far fewer resources for training with deep neural networks in general and CNN in particular. This makes the SIFT method efficient and feasible when working with relatively more minor datasets at the expense of giant data requirements made by the current CNNs. Using SIFT features also yielded a good baseline for comparison with the DL approaches, such that it can be determined whether the additional time complexity and computational cost incurred in the deep model is justified by its performance. In summary, SIFT features present a very persuasive solution for classifying skin lesions due to their robustness, efficiency, and applicability in low-data and low-computational-resource conditions.

### 4.7 Ablation study for LesionNet

We performed an ablation study, where various components of the LesionNet model were systematically dropped and updated to assess their contribution to the final performances. LesionNet is a DL model tailored for skin lesion classification by a few relevant components: SIFT features, and some neural network layers. The modules that have been tested are the SIFT features, convolutional layers, pooling layers, dense layers, batch normalization, and dropout. In this paper, the benchmark Skin Lesion Dataset was evaluated concerning accuracy, F1 score, precision, Kappa, recall, AUC, and MCC.

**Baseline model:** The model includes all components: SIFT features, convolutional-layers, pooling-layers, dense-layers, batch-normalization, and dropout-layers.

**Results:**

- **Removing SIFT features:** accuracy decreased by 9%, precision by 8%, recall by 7%, F1 score by 7%, and AUC by 8%.
- **Removing convolutional layers:** accuracy decreased by 10%, precision by 9%, recall by 11%, F1 score by 10%, and AUC by 9%.
- **Removing pooling layers:** accuracy decreased by 5%, precision by 4%, recall by 6%, F1 score by 5%, and AUC by 5%.
- **Removing dense layers:** accuracy decreased by 8%, precision by 7%, recall by 9%, F1 score by 8%, and AUC by 7%.
- **Removing batch normalization:** accuracy decreased by 4%, precision by 3%, recall by 4%, F1 score by 4%, and AUC by 3%.
- **Removing dropout layers:** accuracy decreased by 6%, precision by 5%, recall by 7%, F1 score by 6%, and AUC by 5%.

**Summary of findings:** each component of LesionNet is crucial for achieving high performance in skin lesion classification. The results indicate that both traditional features (SIFT) and modern DL components are important for building a robust and accurate model.

### 4.8 Performance comparison

It is very important to validate the performance of any proposed model with previously published research works. In this subsection, we have compared the performance of the proposed LesionNet model with other published research works as shown in Table 8. Sikkandar et al. (23) used U-Net transfer learning with 369 images, achieving 78% accuracy. Pour et al. (60) employed C-Means clustering with 65% accuracy. Lopez et al. (61) used the VGG16 model, achieving 82% accuracy. All these models utilize DL models but with no additional feature extraction technique that ultimately results in low performance in terms of accuracy.

**Table 8:** Performance comparison of the proposed LesionNet with previously published research works.

**References**	**Proposed technique**	**Accuracy**	**Limitations**
Sikkandar et al. (23)	UNet	78%	No cross validation, no comparison with SOTA
Codella et al. (62)	Fr-CN	94%	No cross validation, no feature engineering, and no comparison with SOTA
Pour and Seker (60)	CMeans Clusters	65%	No cross validation, no comparison with SOTA
Lopez et al. (61)	ConvNets'	69%	No cross validation, no feature engineering, and no comparison with SOTA
Lopez et al. (61)	VGG-16	82%	No cross validation, no feature engineering, and no comparison with SOTA
**Proposed**	**LesionNet with SIFT features**	**99.28%**	All limitations resolved

Bold indicates the proposed model performance is better than everyone and all limitations are resolved.

The proposed LesionNet framework outperforms both the Grabcut algorithm (23) and CIELAB color space (60) due to its superior feature extraction capabilities. SIFT (Scale-Invariant Feature Transform) is highly effective in detecting and describing local features that are invariant to scaling, rotation, and illumination, making it robust for complex skin lesions. In contrast, Grabcut, primarily a segmentation tool, relies heavily on manual input and lacks the intricate feature extraction needed for accurate lesion classification. CIELAB, focused on color differences, falls short when handling texture and structural variations, which are crucial for skin lesion analysis. By integrating SIFT with a customized CNN, LesionNet provides a more automated and adaptable approach, achieving better accuracy, precision, and robustness, particularly in complex and diverse lesion datasets. This combination of SIFT features and CNN allows for a more comprehensive analysis compared to Grabcut and CIELAB's limited segmentation and color-based methods.

## 5 Conclusion

Most skin cancers look alike and appear very similar to skin injuries and other infections. They are only slightly different in shape or size, thus challenging doctors to diagnose skin lesions in the early stages of development. Besides, many subtypes of skin cancer add to the diagnostic difficulty. Early detection is important because it allows for early intervention and saves many precious lives. To help in tending to these challenges, this study proposes a novel approach for skin lesion diagnosis with visual imagery of skin cancer. The proposed model, LesionNet, integrates SIFT features with a deep convolutional neural network model. SIFT features are used to extract relevant features upon which LesionNet is trained to learn. Through experimental evaluation, the model's efficiency is tested against the HoG method, which turns out to have less satisfactory results. The results, however, were much more in favor of the proposed model, with a resultant accuracy as high as 99.28%. Future research efforts are directed toward expanding this approach for diagnosing various cancer types other than skin cancer to increase its scope of application. As part of future work, expanding the dataset to include a more diverse set of skin lesion images from various ethnicities and geographical regions to generalize the proposed framework. Furthermore, we will deploy LesionNet model in real-world environment to check its reliability with the help of concerned dermatologists.

## Data Availability

The original contributions presented in the study are included in the article/supplementary material, further inquiries can be directed to the corresponding authors.
